# Brain responses and looking behavior during audiovisual speech integration in infants predict auditory speech comprehension in the second year of life

**DOI:** 10.3389/fpsyg.2013.00432

**Published:** 2013-07-16

**Authors:** Elena Kushnerenko, Przemyslaw Tomalski, Haiko Ballieux, Anita Potton, Deidre Birtles, Caroline Frostick, Derek G. Moore

**Affiliations:** ^1^Institute for Research in Child Development, School of Psychology, University of East LondonLondon, UK; ^2^Faculty of Psychology, University of WarsawWarsaw, Poland

**Keywords:** audiovisual speech integration, infants’ brain responses, ERPs, eye-tracking, language development, mismatch

## Abstract

The use of visual cues during the processing of audiovisual (AV) speech is known to be less efficient in children and adults with language difficulties and difficulties are known to be more prevalent in children from low-income populations. In the present study, we followed an economically diverse group of thirty-seven infants longitudinally from 6–9 months to 14–16 months of age. We used eye-tracking to examine whether individual differences in visual attention during AV processing of speech in 6–9 month old infants, particularly when processing congruent and incongruent auditory and visual speech cues, might be indicative of their later language development. Twenty-two of these 6–9 month old infants also participated in an event-related potential (ERP) AV task within the same experimental session. Language development was then followed-up at the age of 14–16 months, using two measures of language development, the Preschool Language Scale and the Oxford Communicative Development Inventory. The results show that those infants who were less efficient in auditory speech processing at the age of 6–9 months had lower receptive language scores at 14–16 months. A correlational analysis revealed that the pattern of face scanning and ERP responses to audiovisually incongruent stimuli at 6–9 months were both significantly associated with language development at 14–16 months. These findings add to the understanding of individual differences in neural signatures of AV processing and associated looking behavior in infants.

## INTRODUCTION

Visual speech cues are known to facilitate speech comprehension when auditory input is ambiguous, for example in a noisy environment, with the shape of the mouth partially indicating the sounds produced (Sumby and Pollack, 1954). Seeing someone speak may improve the comprehension of hard-to-understand passages even when hearing conditions are excellent (for a review see Campbell, 2008). A method for assessing capacities for audiovisual (AV) speech integration (AVSI) in adults and infants is to present simple video clips of people pronouncing syllables (/ba/ or /ga/) including clips where the visual and auditory speech components of the stimuli do not match (Kushnerenko et al., 2008). In these non-matching circumstances the fusion and the combination speech illusions may be perceived, a phenomenon known as the McGurk effect (McGurk and MacDonald, 1976). Of particular interest is what happens when a visual /ga/ and auditory /ba/ are presented together (VgaAba) as these are often fused by adults and perceived as the sound /da/ or /θ/. On the other hand a visual /ba/ dubbed onto auditory /ga/ (VbaAga) is often perceived as the combination /bga/.

Developmental studies of AVSI offer ambiguous results with respect to this phenomenon in infancy. Some behavioral studies indicate that infants as young as 4 months of age can perceive the McGurk “fusion” illusion (Rosenblum et al., 1997; Burnham and Dodd, 2004). Electrophysiological studies further indicate that 5 month-olds process the two kinds of audiovisually incongruent stimuli differently (Kushnerenko et al., 2008), suggesting that these lead to the same “combination” and “fusion” effects as are seen in adults. In this study, the AV mismatch response (AVMMR) was recorded in response to the VbaAga-combination condition but not to the VgaAba-fusion.

On the other hand, Desjardins and Werker (2004) demonstrated that AV integration is not an obligatory process in young infants and that it may require a degree of experience with language before emerging. Further, Massaro (1984) hypothesized that differences between adults and children in AVSI can be explained by different levels of attention to the visual component of the stimuli. For example, the use of visual cues during AV processing of speech is known to be less efficient in children and adults with language-learning disabilities (Norrix et al., 2006, 2007). Also, difficulties in integrating auditory and visual aspects of speech perception have been reported in children with specific language impairment (Pons et al., 2013) and in autism spectrum disorder (ASD; Guiraud et al., 2012; Megnin et al., 2012).

Attention to visual speech cues appears to undergo significant changes over the first year of life. Lewkowicz and Hansen-Tift (2012) demonstrated a developmental shift in visual attention to articulating faces within the first 12 months of life from an initial tendency to look at the eyes rather than the mouth, followed by a marked increase in looking at the mouth, returning to preference for the eyes at 12 months of age. This pattern in attentional shifts may correspond with transitional periods in speech acquisition in infancy. For example, recent studies have demonstrated that visual attention to the eye region at 6 months, but not at 9 and 12 months, is associated with better social and communicative outcomes at the age of 18 months (Schietecatte et al., 2012; Wagner et al., 2013).

Visual attention, specifically during AVSI, has recently been investigated in detail in 6- to 9-month-old infants using the paradigm developed by Kushnerenko, Tomalski, and colleagues (Tomalski et al., 2012; Kushnerenko et al., 2013). In this eye-tracking (ET) paradigm, faces articulating either /ba/ or /ga/ syllables were displayed along with the original auditory syllable (congruent VbaAba and VgaAga), or a mismatched one (incongruent VbaAga and VgaAba). By measuring the amount of looking to the eyes and mouth of articulating faces, it was found that younger infants (6–7 months) may not perceive mismatching auditory /ga/ and visual /ba/ (VbaAga) cues in the same way as adults, that is, as the combination /bga/ (McGurk and MacDonald, 1976) but process these stimuli as a mismatch between separate cues and “reject” them as a source of unreliable information, and therefore allocate less attention to them. Using the same stimuli, Kushnerenko et al. (2013) also found that the AVMMR brain response to these stimuli showed large individual differences between 6 and 9 months of age, and that these differences were strongly associated with differences in patterns of looking to the speaker’s mouth. Interestingly, the amplitude of the AVMMR was inversely correlated with looking time to the mouth, which is consistent with the results found by Wagner et al. (2013). These results suggest that at this age sufficient looking toward the eyes may play a pivotal role for later communicative and language development. Given these results, and the fact that infants as young as 2–5 months of age are able to match auditory and visual speech cues (Kuhl and Meltzoff, 1982; Patterson and Werker, 2003; Kushnerenko et al., 2008; Bristow et al., 2009), we hypothesized that individual differences in visual attention and brain processing of AV speech sounds should predict language development at a later age.

In the current paper we report the results of a follow-up study with infants who at the age of 6- to 9-months completed an AVSI task with matching and mismatching speech cues. AVSI was assessed with both ET and event-related potential (ERP) measures in the same task, reported elsewhere (Tomalski et al., 2012; Kushnerenko et al., 2013). For the present follow-up, infants attended a session when they were 14- to 16-months-old, and their early language and communicative development was assessed using language assessment tests. The sample had been recruited from areas with a high multiple deprivation index with the purpose of recruiting a diverse sample in terms of family socio-economic status (SES) in order to capture a range of abilities. Several studies have indicated that children from low-SES areas have weaker language skills at preschool age (Raizada et al., 2008) and deficits in selective attention related to speech processing, including a reduced ability to filter out irrelevant auditory information (Stevens et al., 2009). We therefore expected a representative proportion of our sample of infants to be at risk of later language related difficulties.

There is now evidence for the existence of early individual differences in how young infants visually scan social stimuli (Kushnerenko et al., 2013). There is also evidence that these individual differences can be predictive of later language (e.g., Young et al., 2009) and communicative development (Wagner et al., 2013). Also, auditory-only speech sound discrimination in 6-month-olds predicts later vocabulary (e.g., Tsao et al., 2004). Given this evidence we have sought to establish whether individual differences in AV speech processing at 6- to 9-months of age predict language development at 14- to 16 months. In particular we measured the neural responses and the amount of time spent fixating the eyes and the mouth of articulating faces with mismatching AV speech cues. We hypothesized that the pattern of visual attention to incongruent AV speech cues in infancy and sensitivity to AV mismatch as reflected by brain responses might be a significant predictor of receptive and expressive language in toddlers.

## MATERIALS AND METHODS

### PARTICIPANTS

All 37 infants had previously participated in an ET AV task (Tomalski et al., 2012) when aged between 6 and 9 months (10 were boys; the mean age was 33.5 weeks, SD = 2.8 weeks). Twenty-two of these infants (6 boys, mean age 30.7 weeks, SD = 4.3 weeks) also participated in an ERP AV task (Kushnerenko et al., 2013). The birth weight of infants and gestational ages were in the normal range (mean weight 3377.6 g; mean gestational age 39.59 weeks). The average total income of the families was £52,401 and ranged from £4,800 to £192,000, which represents a large income range (see **Table 1**). The age range for this study was chosen because neural signatures of auditory processing demonstrate different rates of maturation during this age period, with some 6 month-olds showing a more mature ERP pattern and some 9 month-olds a less mature one (Kushnerenko et al., 2002). The study was approved by the local ethics committee and conformed to the Declaration of Helsinki. Prior to the study parents gave written informed consent for their child’s participation.

**Table 1:** Demographic characteristics of the higher AC-PLS and lower AC-PLS groups of infants (standard deviation).

Measure		All infants (*n* = 37)	Lower AC-PLS (*n* = 19)	Higher AC-PLS (*n* = 18)
Age at second		65.49	65.05	65.65
session (weeks)		(3.24)	(2.62)	(3.67)
Gender	Female	27 10	13 6	14 4
	Male			
Gestational age		39.59	40.00	39.24
(weeks)		(1.87)	(1.81)	(1.89)
Birth weight		3377.6	3400.17	3358.33
(grams)		(413.9)	(366.9)	(458.47)
Average income		52,401	43,002	60,518
£		(43,062)	(35,901)	(47,732)
Mother SOC	(1)	47.5%	50.0%	63.6%
	(2)	17.5%	22.2%	13.6%
	(3)	25.0%	27.7%	22.7%

### LANGUAGE ASSESSMENT AT 14–16 MONTHS

Infants were assessed individually using the PreSchool Language Scale-4 (PLS-4; Zimmerman et al., 2002) between 14 and 16 months (mean = 14.7, SD = 0.7). The PLS-4 is a norm-referenced test of receptive and expressive language ability for ages from birth to 6 years and 11 months. The test consists of a picture book and manipulative toys designed to engage a child in order to elicit responses to test items. The test gives two standardized sub-scales, auditory comprehension (AC) and expressive communication (EC), and a total score. During the follow-up parents were also asked to complete the Oxford Communicative Development Inventory (OCDI, a UK adaptation of the MacArthur-Bates CDI). The OCDI is a tool for assessing the development of receptive and productive vocabulary through parental report and is typically used with children aged from about 11–26 months (Hamilton et al., 2000). Basic demographic information on family income, parental education and occupation was collected from the primary caregivers (see **Table 1**) via a study-designed questionnaire (Tomalski et al., 2013).

### EYE-TRACKING TASK AT 6–9 MONTHS

Infants were seated on their caregiver’s lap in a dimly lit room. They were seated approximately 60 cm in front of a Tobii T120 eye-tracker monitor (17″ diameter, screen refresh rate 60 Hz, ET sampling rate of 120 Hz, spatial accuracy 0.5°). Prior to the experiment each infant’s eye movements were calibrated using a five-point routine in order to ensure positional validity of gaze measurements. At least 50% of samples were recorded from each infant during each trial. The parent’s view of the stimulus monitor was obscured to prevent interference with the infant’s looking behavior. Eye movements were monitored continuously during each recording. Every infant observed a total of ten trials. Before each trial, infants’ attention was directed to the screen by colorful animations with sound, and these were terminated as soon as the infant fixated them. For more details on the ET task see Tomalski et al. (2012).

The stimuli were two video clips of female native English speakers articulating /ba/ and /ga/ syllables and two incongruent pairs which were created from the original AV stimuli by dubbing the auditory /ba/ onto a visual /ga/ (VgaAba) and vice versa (VbaAga). Sound onset in each clip was 360 ms from stimulus onset, and auditory syllable duration was 280–320 ms. The total duration of one AV stimulus was 760 ms. For more information on the stimuli see Kushnerenko et al. (2008). Each trial contained 10 repetitions of one type of stimulus and the trial duration was 7600 ms (760 ms × 10). The entire sequence lasted approximately 2 min.

### EVENT-RELATED POTENTIAL STUDY AT 6–9 MONTHS

The paradigm and stimuli for this task were the same as in Kushnerenko et al. (2008).

The same AV stimuli as in the ET study were presented in a pseudorandom order. Videos were displayed on a CRT monitor (30 cm diameter, 60 Hz refresh rate) with a black background. The infants were seated on the caregiver’s lap in an acoustically and electrically shielded booth. They were seated at a distance of 80 cm from the monitor. At that distance the faces on the monitor were approximately life size. Sounds were presented at about a 65 dB level via two loudspeakers behind the screen. The recording time varied from 4 to 6 min, depending on each infant’s attention to the stimuli. The behavior of the infants was videotaped and coded off-line for electroencephalography (EEG) artifact rejection.

High-density EEG was recorded with a 128-channel Hydrocel Sensor Net (EGI Inc.) referenced to the vertex (Tucker, 1993). The EEG signal was amplified, digitized at 500 Hz, and band-pass filtered from 0.1 to 200 Hz. The signal was off-line low-pass filtered at 30 Hz and segmented into epochs starting 100 ms before and ending 1,000 ms after the AV stimulus onset. Channels contaminated by eye or motion artifacts were rejected manually, and trials with more than 20 bad channels were excluded. In addition, video recordings of the infants’ behavior were coded frame-by-frame, and trials during which the infant did not attend to the face were excluded from further analysis. Following artifact rejection, the average number of trials for an individual infant accepted for further analysis was 37.4 for /ba/, 36.7 for /ga/, 37.6 for VgaAba, and 37.8 for VbaAga. Although uncommon for adult ERP studies, this number of accepted trials has proven to be sufficient in infant studies (Dehaene-Lambertz and Dehaene, 1994; Friederici et al., 2007; Kushnerenko et al., 2008; Bristow et al., 2009; Guiraud et al., 2011).

Artifact-free segments were re-referenced to the average reference and then averaged for each infant within each condition. A baseline correction was performed by subtracting mean amplitudes in the 260–360 ms window from the video onset (i.e., immediately before the sound onset) to minimize the effects of any ongoing processing from the preceding stimulus. For the statistical analyses we bilaterally defined channel groups: frontal (area between Fp1, F3, and Fz on the left and symmetrical on the right), central (area between F3, C3, and Cz on the left and symmetrical on the right), occipital (area between O1, P3, and Pz on the left and symmetrical on the right) and temporo-parietal (covering area between P3 and left mastoid and P4 and the right mastoid). The analyses were conducted on mean amplitudes within the time window between 290 and 390 ms from the sound onset for AVMMR (Kushnerenko et al., 2008) and between 140 to 240 ms from the sound onset for infantile P2 (Kushnerenko et al., 2007). The correlation analysis was performed for the frontal and central ERP mean amplitudes and looking time to the eyes and mouth as a percentage of total looking time to the face in both audiovisually mismatching conditions VbaAga and VgaAba. Partial correlations controlled for the age at the first session, total family income, and maternal occupation. The last two variables were taken as indicators of SES of the family, and have been previously found to be associated with the power of frontal gamma oscillations (Tomalski et al., 2013).

## RESULTS

Pearson correlations were computed in order to determine whether neural or behavioral signatures of AV processing at 6–9 months, specifically the processing of a mismatch between auditory and visual speech cues, is associated with language outcome at 14–16 months of age. In this analysis we partialled out age at first assessment, total family income, and maternal occupation. These factors are known to contribute to individual differences in language outcomes, and we wanted to examine how well early AVSI responses can predict language outcomes, having controlled for these potential mediating variables.

### ASSOCIATIONS BETWEEN ATTENTION TO AUDIOVISUAL SPEECH AT 6–9 MONTHS AND LANGUAGE DEVELOPMENT AT 14–16 MONTHS

**FIGURE 1:**
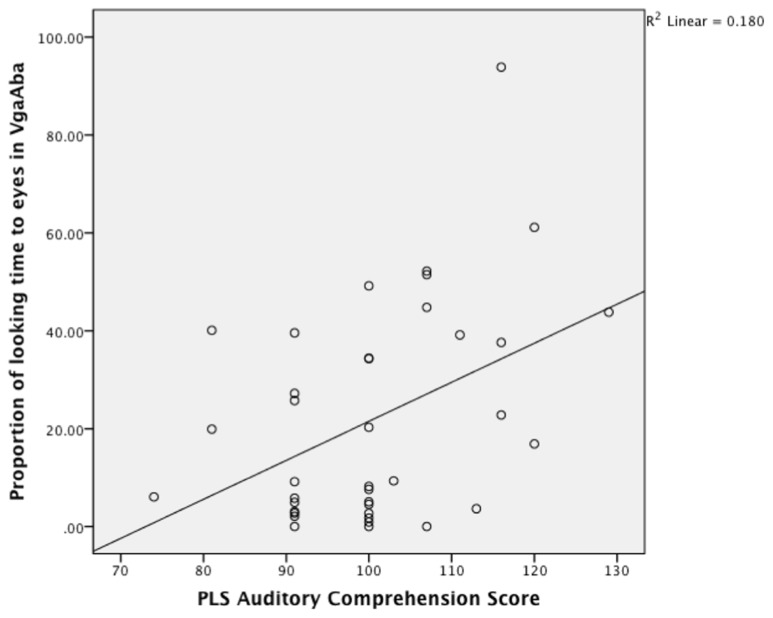
**A scatterplot showing the relationship between looking time to eyes in VgaAba condition at 6–9 months of age and PLS auditory comprehension scale at 14–16 months**.

Partial Pearson correlations confirmed that PLS-4 AC scores were significantly negatively correlated with looking time to the mouth in the VbaAga condition (**Table 2**), and positively correlated with looking time to the eyes in the VgaAba condition (see also **Figure 1**). These results indicate a similar tendency for both incongruent AV conditions: infants who received higher scores for their language development had shorter looking times to the mouth area and/or longer looking times to the eyes when they encountered AV mismatch.

**Table 2:** Partial correlations for PLS-4 and Oxford CDI scores at 14–16 months and eye-tracking and ERP measurements at 6–9 months of age (partial-*r* and p).

	Looking time to eyes in VbaAga	Looking time to mouth in VbaAga	Looking time to eyes in VgaAba	Looking time to mouth in VgaAba	Frontal left P2 amplitude	Frontal right P2 amplitude
Oxford CDI comprehension	0.09	0.01	0.32	-0.27	-0.06	-0.10
	0.59	0.94	0.05	0.12	0.81	0.66
Oxford CDI production	0.01	-0.19	0.41	-0.29	-0.18	-0.04
	0.96	0.26	0.01^*^	0.09	0.46	0.88
PLS auditory comprehension	0.31	-0.34	0.35	-0.16	-0.68	-0.48
	0.07	0.04^*^	0.03^*^	0.35	0.001^*^	0.04^*^
PLS expressive communication	-0.20	0.05	-0.15	0.03	-0.09	-0.16
	0.25	0.74	0.37	0.87	0.74	0.55

* p<0.05.

As **Figure 1** demonstrates, the longer infants looked at the eyes in the VgaAba condition, the better their AC was 1 year later. In addition, there were significant correlations between looking time to the eyes in this condition and the Oxford CDI productive vocabulary (OCDI) score (partial-*r* = 0.42, *p* = 0.01), as well as a marginally significant association with OCDI comprehension score (partial-*r* = 0.32, *p* = 0.06).

### ASSOCIATIONS BETWEEN ERP MEASURES OF AV PROCESSING AT 6–9 MONTHS AND LANGUAGE DEVELOPMENT AT 14–16 MONTHS

**FIGURE 2:**
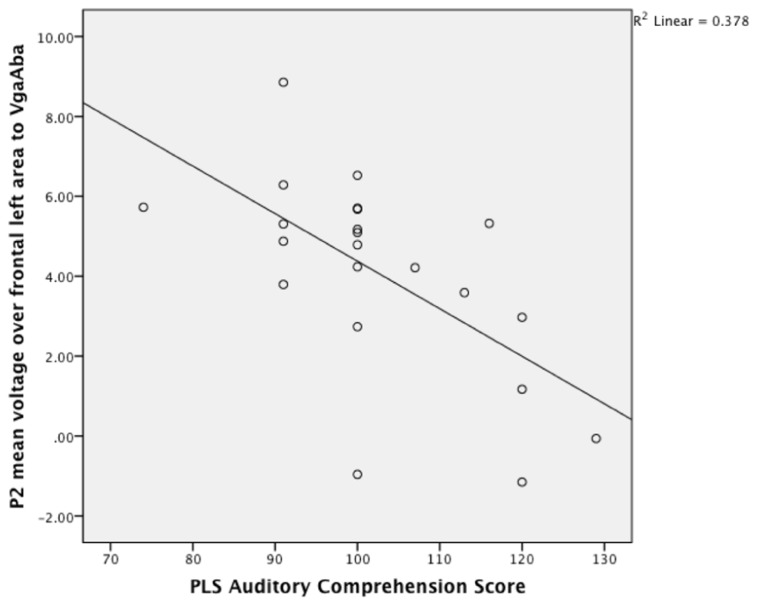
**A scatterplot showing the relationship between the mean P2 voltage over the left frontal area (140–240 ms) in response to VgaAba at 6–9 months of age and PLS auditory comprehension scale at 14–16 months**.

Given the result that lower language scores at 14–16 months were associated with longer looking time to the mouth area, we also expected an association with larger frontal P2 amplitudes in response to the VgaAba-fusion condition. In a previous study we have found an association between looking time to the mouth and frontal P2 amplitude (Kushnerenko et al., 2013). Indeed, partial correlation coefficients were significant (partial-*r* = -0.68, *p* = 0.001, partial-*r* = 0.48, *p* = 0.04) for PLS-4 AC scores and the amplitude of the infantile P2 over frontal areas in response to the same stimulus (VgaAba; see **Figure 2**). It should be noted that correlations for the mean voltage over the frontal area were negative, which indicates that larger P2 amplitudes are associated with poorer language comprehension.

For illustration purposes, the participants were median split into low and high groups on the basis of AC (see **Table 1** for the demographics profile of these two groups). Note that although they appear to differ in income, there were no significant differences between these groups on demographic measures.

**FIGURE 3:**
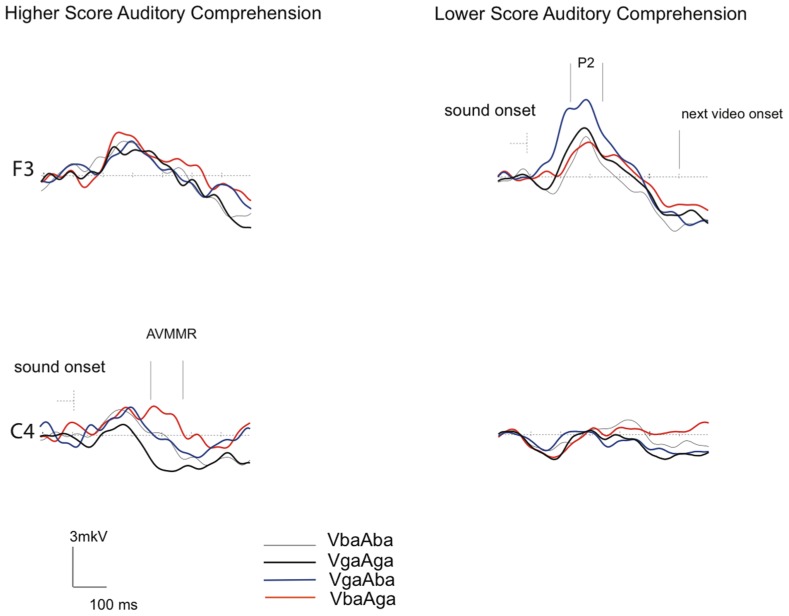
**Grand-averaged ERP responses to congruent and incongruent AV stimuli in 6–9 month-old infants with higher and lower AC-PLS scores at the follow-up age.** ERP responses plotted for VbaAba (thin grey), VbaAga (orange), VgaAba (blue), and VgaAga (black) time-locked to the sound onset. Selected channels are shown according to 10–20 system.

**Figure 3** demonstrates that while the P2 amplitude in response to congruent AV /ba/ and /ga/ stimuli is of about the same amplitude in both groups, in response to incongruent VgaAba stimuli it appears to be larger over the frontal area in infants with lower AC-PLS scores (F3 channel). In addition, although no significant associations were found between language outcome and the amplitude of the AVMMR, this brain response to incongruent VbaAga was only observed in the higher AC-PLS group of infants.

**FIGURE 4:**
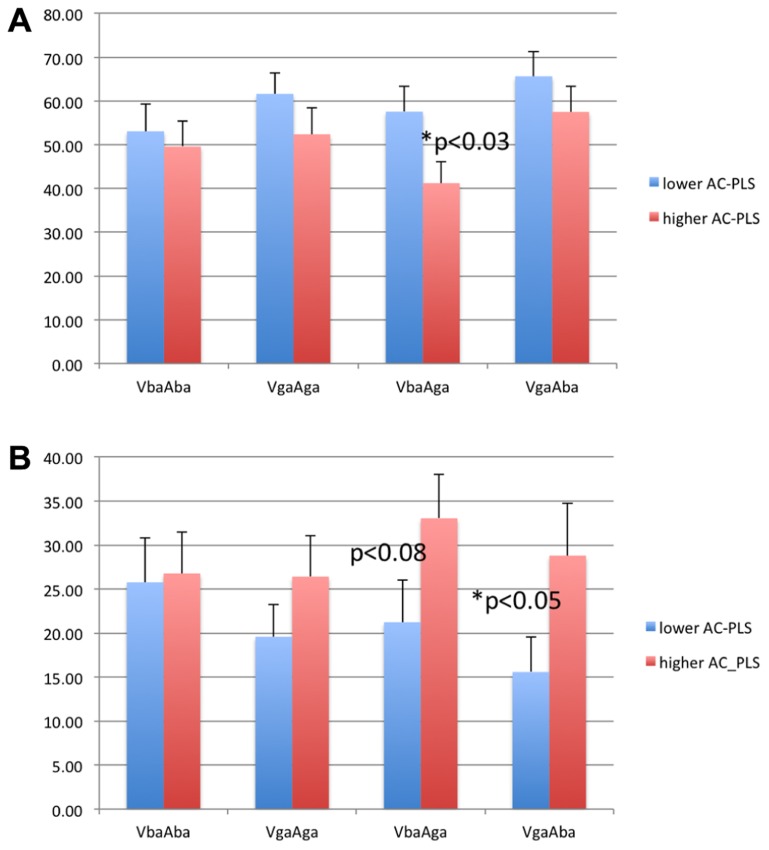
**Looking time to the mouth (A) and eyes **(B)** as percentage of looking time to the face in 6–9 month-old infants with higher and lower AC-PLS scores at the follow-up age**.

**Figure 4** shows the percentage of looking time to the eyes and mouth in both groups of infants. The subgroup of infants with higher AC-PLS scores showed generally longer looking times to the eyes and shorter looking times to the mouth. However, the difference between groups was significant only for the incongruent conditions (two-sample *t* test, *p* < 0.03 for VbaAga-Mouth, and *p* < 0.05 for VgaAba-Eyes).

## DISCUSSION

The aim of the present study was to investigate whether individual differences in neural and behavioral markers of AV processing in infancy might be indicative of later language development. The follow-up assessment revealed significant associations between ET and EEG measures at 6–9 months and language development measures at 14–16 months of age. Specifically, infants who spent a longer time watching the eyes of a female face when auditory and visual speech cues did not match performed better on the AC scale of the Preschool Language Scale (PLS-4). The level of functioning on the vocabulary scale of the Oxford CDI was also consistent with this result and showed a significant association with looking time to the eyes in the mismatched condition. In addition, infants who had higher AC-PLS scores appeared to look less at the mouth during presentation of the saliently mismatched condition (VbaAga).

As shown previously, this pattern of responses where infants show shorter looking times to the mouth when auditory and visual stimuli are mismatched is a transitional phase in development between the ages of 6 and 9 months. In an earlier study Tomalski et al. (2012) found a positive association between age and the amount of looking at the mouth for mismatched AV speech cues.

By the time infants reach 9 months of age, they clearly show longer looking to the mouth while watching incongruent AV speech cues compared with the congruent syllables (Tomalski et al., 2012). At the same time the neural AVMMR in response to AV mismatch significantly decreases in amplitude, indicating that this signature of AV mismatch processing in infancy is transitory (Kushnerenko et al., 2013).

Nardini et al. (2010) proposed that not integrating sensory cues might be adaptive for younger children because they must learn not only to combine cues but also to establish whether these cues are reliable, and whether some cues should be ignored. The developmental pattern observed between 6 and 9 months of age in ET (Tomalski et al., 2012) is consistent with this idea: shorter looking times to the mouth in the mismatched condition indicate that younger infants ignored unreliable and confusing visual cues.

The results of the present study indicate that the ability to detect a mismatch between visual and auditory cues during this transitional phase might be indicative of AC in the second year of life. This may imply that infants who spent longer time watching mouth articulation during mismatched AV trials might have difficulties with recognizing the auditory component and therefore might seek more information from lip movements. By contrast, toddlers with higher AC scores may have correctly recognized the speech sound during the ET and ERP tasks at 6–9 months, but did not find it helpful to attend to the distracting and unreliable lip movements. Thus, longer looking times to the eyes during mismatched AV trials in these infants may indicate that they were searching for additional social cues to resolve the ambiguity of these stimuli.

This assumption is consistent with a recent study that shows visual attention to the eye region (measured using ET at 6 months) to be associated with better social outcome at the age of 18 months, as measured by the Communication and Symbolic Behavior Scales (Wagner et al., 2013; see also Schietecatte et al., 2012). Interestingly, the association was significant for younger infants (6-month-olds) but not for 9- and 12-month-olds. This finding illustrates once again that the pattern of visual attention in infants largely depends on their maturational level (Lewkowicz and Hansen-Tift, 2012; Kushnerenko et al., 2013).

On the other hand, another longitudinal study yielded the opposite pattern of results: infants with longer fixation on the mouth demonstrated better expressive language skills later on (Young et al., 2009). However, the group of infants tested in the study by Young and colleagues had a higher familial risk of ASD and the design of the study was different, with infants only seeing congruent live mother–infant interaction and no confusing AV information. In the present study, differences in looking behavior between infants with higher and lower AC scores were only significant for incongruent AV conditions (VbaAga and VbaAga). Therefore, the results of Young and colleagues (2009) seem to demonstrate a different phenomenon and are not comparable with those of the current study. In addition, in the study of Young and colleagues (2009) the correlations were found for the expressive language score and not for AC. We propose therefore that attention to the mouth is more important for the development of expressive language because it facilitates imitation and is useful for learning how to articulate particular speech sounds (Howard and Messum, 2011). On the other hand, AC abilities are likely to be more related to the accuracy of auditory processing in young infants. Attention to the eyes then may assist in learning new object labels. Infants increasingly use referential gaze as a cue to direct their looking toward an object that is being named (e.g., Gliga and Csibra, 2009) and benefit from referential gaze in their language learning (Houston-Price et al., 2006).

In the present study, significant associations were also found between receptive language score at 14–16 months and ERP measures of AV processing at 6–9 months of age. A larger amplitude of the frontal P2 was found in response to the incongruent VgaAba stimulus in a subgroup of infants with lower AC score at the follow-up age. Larger P2 amplitudes (positive over frontal and negative over occipital areas) to incongruent AV stimuli have previously been observed in infants who spent longer time attending to lip articulations than to eyes (Kushnerenko et al., 2010, 2013).The increased P2 may have contributions from the activity of visual areas, therefore demonstrating that infants who look longer at the mouth might be processing visual cues more intensively than auditory ones. In the present follow-up study, both the increased frontal P2 amplitude and longer looking time to the mouth during the mismatch VbaAga condition in infancy were associated with less advanced AC later in development. One possible explanation for this could be that infants who have less accurate or less mature auditory speech processing at the age of 6–9 months rely more on using visual cues when ambiguous speech stimuli are presented. This pattern of results may indicate that a visual-over-auditory bias in sensory processing of speech cues at 6 months of age can be predictive of less advanced auditory speech comprehension at the age of 14–16 months.

To summarize, in the present study the larger frontal P2 amplitudes to the ambiguous AV stimuli were associated with lower AC scores on language scales in 14–16 month-old toddlers. In addition, there was a significant association between longer looking times to the eyes than to the mouth in the incongruent conditions and the higher AC score (and the opposite tendency for longer looking times to the mouth). These findings provide important evidence that early markers of infants’ visual attention relate not only to their social development (Schietecatte et al., 2012; Wagner et al., 2013) but also to their later language development. The current results also demonstrate that early electrophysiological indices of AV speech processing are indicative of language comprehension in the second year of life.

## Conflict of Interest Statement

The authors declare that the research was conducted in the absence of any commercial or financial relationships that could be construed as a potential conflict of interest.

## References

[B1] Bristow D. Dehaene-Lambertz G. Mattout J. Soares C. Gliga T. Baillet S. (2009). Hearing faces: how the infant brain matches the face it sees with the speech it hears. *J. Cogn. Neurosci.* 21 905–921 10.1162/jocn.2009.21076 18702595

[B2] Burnham D. Dodd B. (2004). Auditory-visual speech integration by prelinguistic infants: perception of an emergent consonant in the McGurk effect. *Dev. Psychobiol.* 45 204–220 10.1002/dev.20032 15549685

[B3] Campbell R. C.-P. (2008). The processing of audio-visual speech: empirical and neural bases. *Philos. Trans. R. Soc. Lond. B Biol. Sci.* 363 1001–1010 10.1098/rstb.2007.2155 17827105 PMC2606792

[B4] Dehaene-Lambertz G. Dehaene S. (1994). Speed and cerebral correlates of syllable discrimination in infants. *Nature* 28 293–294 10.1038/370292a0 8035876

[B5] Desjardins R. N. Werker J. F. (2004). Is the integration of heard and seen speech mandatory for infants? *Dev. Psychobiol.* 45 187–203 10.1002/dev.20033 15549681

[B6] Friederici A. D. Friedrich M. Christophe A. (2007). Brain responses in 4-month-old infants are already language specific. *Curr. Biol.* 17 1208–1211 10.1016/j.cub.2007.06.011 17583508

[B7] Gliga T. Csibra G. (2009). One-year-old infants appreciate the referential nature of deictic gestures and words. *Psychol. Sci.* 20 347–353 10.1111/j.1467-9280.2009.02295.x 19207689

[B8] Guiraud J. A. Kushnerenko E. Tomalski P. Davies K. Ribeiro H. Johnson M. H. (2011). Differential habituation to repeated sounds in infants at high risk for autism. *Neuroreport* 22 845–849 10.1097/WNR.0b013e32834c0bec 21934535

[B9] Guiraud J. A. Tomalski P. Kushnerenko E. Ribeiro H. Davies K. Charman T. (2012). Atypical audiovisual speech integration in infants at risk for autism. *PLoS ONE* 7:e36428 10.1371/journal.pone.0036428 PMC3352915 22615768

[B10] Hamilton A. Plunkett K. Schafer G. (2000). Infant vocabulary development assessed with a british communicative development inventory: lower scores in the UK than the USA. *J. Child Lang*. 27 689–705 11089344 10.1017/s0305000900004414

[B11] Houston-Price C. Plunkett K. Duffy H. (2006). The use of social and salience cues in early word learning. *J. Exp. Child Psychol.* 95 27–55 10.1016/j.jecp.2006.03.006 16677668

[B12] Howard I. S. Messum P. (2011). Modeling the development of pronunciation in infant speech acquisition learning to pronounce. *Motor Control* 1 85–117 21339516 10.1123/mcj.15.1.85

[B13] Kuhl P. K. Meltzoff A. N. (1982). The bimodal perception of speech in infancy. *Science* 218 1138–1141 10.1126/science.7146899 7146899

[B14] Kushnerenko E. Čeponiené R. Balan P. Fellman V. Näätänen R. Huotilainen M. (2002). Maturation of the auditory change-detection response in infants: a longitudinal ERP study. *Neuroreport* 13 1843–1848 12395076 10.1097/00001756-200210280-00002

[B15] Kushnerenko E. Teinonen T. Volein A. Csibra G. (2008). Electrophysiological evidence of illusory audiovisual speech percept in human infants. *Proc. Natl. Acad. Sci. U.S.A.* 105 11442–11445 10.1073/pnas.0804275105 18682564 PMC2516214

[B16] Kushnerenko E. Tomalski P. Ballieux H. Ribeiro H. Potton A. Axelsson E. L. (2010). Audiovisual speech integration: visual attention to articulation affects brain responses in 6-9 month old infants. *Paper presented at EPS/SEPEX * 15–17 April 2010 Granada Spain 10.1016/j.wocn.2009.04.002

[B17] Kushnerenko E. Tomalski P. Ballieux H. Ribeiro H. Potton A. Axelsson E. L. (2013). Brain responses to audiovisual speech mismatch in infants are associated with individual differences in looking behaviour. *Eur. J. Neurosci.* 10.1111/ejn.12317 23889202

[B18] Kushnerenko E. Winkler I. Horváth J. Näätänen R. Pavlov I. Fellman V. (2007). Processing acoustic change and novelty in newborn infants. *Eur. J. Neurosci.* 26 265–274 10.1111/j.1460-9568.2007.05628.x 17573923

[B19] Lewkowicz D. J Hansen-Tift A. M. C. -P. (2012). Infants deploy selective attention to the mouth of a talking face when learning speech. *Proc. Natl. Acad. Sci. U.S.A.* 109 1431–1436 10.1073/pnas.1114783109 22307596 PMC3277111

[B20] Massaro D. W. (1984). Children’s perception of visual and auditory speech. *Child Dev.* 55 1777–1788 10.2307/1129925 6510054

[B21] McGurk H. MacDonald J. (1976). Hearing lips and seing voices. *Nature* 264 746–748 10.1038/264746a0 1012311

[B22] Megnin O. Flitton A. Jones C. R. G. De Haan M. Baldeweg T. Charman T. (2012). Audiovisual speech integration in autism spectrum disorders: ERP evidence for atypicalities in lexical-semantic processing. *Autism Res.* 5 39–48 10.1002/aur.231 22162387 PMC3586407

[B23] Nardini M. Bedford R. Mareschal D. (2010). Fusion of visual cues is not mandatory in children. *Proc. Natl. Acad. Sci. U.S.A.* 107 17041–17046 10.1073/pnas.1001699107 20837526 PMC2947870

[B24] Norrix L. W. Plante E. Vance R. (2006). Auditory-visual speech integration by adults with and without language-learning disabilities. *J. Commun. Disord.* 39 22–36 10.1016/j.jcomdis.2005.05.003 15950983

[B25] Norrix L. W. Plante E. Vance R. Boliek C. A. (2007). Auditory-visual integration for speech by children with and without specific language impairment. *J. Speech Lang. Hear. Res.* 50 1639–1651 10.1044/1092-4388(2007/111) 18055778

[B26] Patterson M. L. Werker J. F. (2003). Two-month-old infants match phonetic information in lips and voice. *Dev. Sci.* 6 191–196 10.1111/1467-7687.00271

[B27] Pons F. Andreu L. Sanz-Torrent M. Buil-Legaz L. Lewkowicz D. J. (2013). Perception of audio-visual speech synchrony in Spanish-speaking children with and without specific language impairment. *J. Child Lang.* 40 687–700 10.1017/S0305000912000189 22874648 PMC3954717

[B28] Raizada R. D. S. Richards T. L. Meltzoff A. Kuhl P. K. (2008). Socioeconomic status predicts hemispheric specialisation of the left inferior frontal gyrus in young children. *Neuroimage* 40 1392–1401 10.1016/j.neuroimage.2008.01.021 18308588 PMC2679945

[B29] Rosenblum L. D. Schmuckler M. A. Johnson J. A. (1997). The McGurk effect in infants. *Percept. Psychophys.* 59 347–357 10.3758/BF03211902 9136265

[B30] Schietecatte I. Roeyers H. Warreyn P. (2012). Can infants’ orientation to social stimuli predict later joint attention skills? Br. *J. Dev. Psychol*. 30 267–282 10.1111/j.2044-835X.2011.02039.x 22550948

[B31] Stevens C. Lauinger B. Neville H. (2009). Differences in the neural mechanisms of selective attention in children from different socioeconomic backgrounds: an event-related brain potential study. *Dev. Sci.* 12 634–646 10.1111/j.1467-7687.2009.00807.x 19635089 PMC2718768

[B32] Sumby W. H. Pollack I. (1954). Visual contribution to speech intelligibility in noise. *J. Acoust. Soc. Am.* 26 212–215 10.1121/1.1907309

[B33] Tomalski P. Moore D. G. Ribeiro H. Axelsson E. L. Murphy E. l. Karmiloff-Smith A. (2013). Socio-economic status and functional brain development – associations in early infancy. *Dev. Sci.* 10.1111/desc.12079 24033573

[B34] Tomalski P. Ribeiro H. Ballieux H. Axelsson E. L. Murphy E. Moore D. G. (2012). Exploring early developmental changes in face scanning patterns during the perception of audiovisual mismatch of speech cues. *Eur. J. Dev. Psychol. * 1–14. 10.1080/17405629.2012.728076

[B35] Tsao F.-M. Liu H.-M. Kuhl P. K. (2004). Speech perception in infancy predicts language development in the second year of life: a longitudinal study. *Child Dev.* 75 1067–1084 10.1111/j.1467-8624.2004.00726.x 15260865

[B36] Tucker D. M. (1993). Spatial sampling of head electrical fields: the geodesic sensor net. *Electroencephalogr. Clin. Neurophysiol.* 87 154–163 7691542 10.1016/0013-4694(93)90121-b

[B37] Wagner J. B. Luyster R. J. Yim J. Y. Tager-Flusberg H. Nelson C. A. (2013). The role of early visual attention in social development. *Int. J. Behav. Dev.* 37 118–124 10.1177/0165025412486064 PMC4606456 26478642

[B38] Young G. S. Merin N. Rogers S. J. Ozonoff S. (2009). Gaze behavior and affect at 6 months: predicting clinical outcomes and language development in typically developing infants and infants at risk for autism. *Dev. Sci.* 12 798–814 10.1111/j.1467-7687.2009.00833.x 19702771 PMC2732664

[B39] Zimmerman I. Steiner V. Pond R. (2002). *Preschool Language Scale*, 4th Edn. San Antonio: The Psychological Corporation

